# Bipolar Effects in Photovoltage of Metamorphic InAs/InGaAs/GaAs Quantum Dot Heterostructures: Characterization and Design Solutions for Light-Sensitive Devices

**DOI:** 10.1186/s11671-017-2331-2

**Published:** 2017-10-05

**Authors:** Sergii Golovynskyi, Luca Seravalli, Oleksandr Datsenko, Oleksii Kozak, Serhiy V. Kondratenko, Giovanna Trevisi, Paola Frigeri, Enos Gombia, Sergii R. Lavoryk, Iuliia Golovynska, Tymish Y. Ohulchanskyy, Junle Qu

**Affiliations:** 1 College of Optoelectronic Engineering, Key Laboratory of Optoelectronic Devices and Systems of Ministry of Education and Guangdong Province, Shenzhen University, Shenzhen, 518060 People’s Republic of China; 2 Institute of Semiconductor Physics, National Academy of Sciences, Kyiv, 03028 Ukraine; 3 Institute of Materials for Electronics and Magnetism, CNR-IMEM, 43100 Parma, Italy; 4 Department of Physics, Taras Shevchenko National University of Kyiv, Kyiv, 01601 Ukraine

**Keywords:** Nanostructure, Quantum dot, Metamorphic, InAs/InGaAs, Photovoltage, Photoconductivity, Photoluminescence, Defects

## Abstract

The bipolar effect of GaAs substrate and nearby layers on photovoltage of vertical metamorphic InAs/InGaAs in comparison with pseudomorphic (conventional) InAs/GaAs quantum dot (QD) structures were studied. Both metamorphic and pseudomorphic structures were grown by molecular beam epitaxy, using bottom contacts at either the grown *n* ^+^-buffers or the GaAs substrate. The features related to QDs, wetting layers, and buffers have been identified in the photoelectric spectra of both the buffer-contacted structures, whereas the spectra of substrate-contacted samples showed the additional onset attributed to EL2 defect centers. The substrate-contacted samples demonstrated bipolar photovoltage; this was suggested to take place as a result of the competition between components related to QDs and their cladding layers with the substrate-related defects and deepest grown layer. No direct substrate effects were found in the spectra of the buffer-contacted structures. However, a notable negative influence of the *n* ^+^-GaAs buffer layer on the photovoltage and photoconductivity signal was observed in the InAs/InGaAs structure. Analyzing the obtained results and the performed calculations, we have been able to provide insights on the design of metamorphic QD structures, which can be useful for the development of novel efficient photonic devices.

## Background

In the last two decades, composite materials containing semiconductor nanostructures have found great use in photonic applications due to light sensitivity, ease and low cost of fabrication, spectral tunability, and highly efficient emission with short lifetime [1–5]. In(Ga)As quantum dot (QD) heterostructures is an important class of infrared-sensitive nanostructures, which has been widely employed in various photonic devices, such as lasers [2, 6], single-photon sources [7, 8], photodetectors [9–13], and solar cells [14–16]. Numerous investigations have been devoted to improve the photoelectric properties of such light-sensitive devices. For example, the photosensitivity range can be extended via the excitation through intermediate bandgap [17, 18] or multiple exciton generation [19, 20], so that the power conversion efficiencies of QD-based solar cells can exceed in theory the limits of single-bandgap solar cells [21]. The methods like strain-balancing [22] and misfit management technique [23] as well as the thermal annealing [24] are used to reduce strains in these structures, operating the working range [25] as well as increasing the photoresponse due to the suppression of strain-related defects [26] that can act as recombination centers.

An efficient method for the strain reduction is based on the growth of an InGaAs metamorphic buffer (MB) instead of the conventional GaAs one. As a result, InAs/InGaAs QD structures have attracted much interest in last decade [27–29]. By growing the QDs on the InGaAs MB, one can observe essential differences in the formation process and QD optical properties compared with conventional ones in GaAs matrix [25, 30–33]. For example, the InGaAs confining layer reduces the lattice mismatch between QDs and buffer and, hence, strains in QDs. As a result, the bandgap of InAs is reduced and a significant increase in the emission wavelength is observed [34]. Application of the MB as a confining material allows to shift its value into the telecommunication window at 1.3 and 1.55 μm [28, 29, 35, 36].

As well, there have been hopeful attempts to apply the photoelectric properties of the metamorphic InAs QD structures on the design of such light-sensitive devices as metamorphic infrared photodetectors [11–13] and solar cells [37–39]. Some studies were carried out to develop structure design [25, 31–33] and other ones to improve photoelectric properties [39, 40]. Investigations are going on to reduce the strains in the heterostructures [34, 41], as this leads to a substantial improvement in the photocurrent density and spectral response of solar cells [39, 40] as well as in the photoemission efficiency of such structures [29, 32, 42].

Development of the light-sensitive devices requires in-depth study of the photoelectric properties. Photovoltage (PV) or photoconductivity (PC) studies is an ideal tool for the determination of the photoresponse as function of light energy, transitions between levels, carrier transport, and operating range of the device [10, 43, 44]. However, despite that some studies of the photoelectric properties of structures with metamorphic InAs QDs have been performed in last years [37–39, 43], full aspects of the photoresponse mechanism still remain unclear, as along with the influence of the MB on the properties of the nanostructures. In particular, effects of the GaAs substrate and related interfaces on the photoelectric spectra of InAs/InGaAs/GaAs QD structures have not been explored in details. Although significant efforts are devoted to avoid the substrate influence, the photoresponse is affected by both the substrate and nearby layers grown by molecular beam epitaxy (MBE). Thus, while the applied contact geometry is to retain the bottom layers and substrate electrically inactive, a notable negative effect of them on PV and photocurrent has been detected by us in a previous investigation [43]. Very recently, we compared the photoelectric properties of the metamorphic InAs/In_0.15_Ga_0.85_As QD structure with those of a standard InAs/GaAs QD one and found that the photocurrent of metamorphic InAs/In_0.15_Ga_0.85_As heterostructures was not affected by levels related to defects in the vicinity of QD [45]. Furthermore, it has been concluded that efficient photonic devices at 1.3 μm can be developed with similar nanostructures as an active material.

**Fig. 1:**
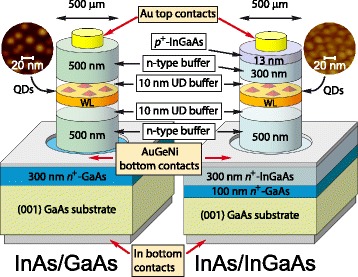
(Color online) schematics of the metamorphic InAs/In_0.15_Ga_0.85_As/*si*-GaAs (right) and InAs/GaAs/*si*-GaAs (left) QD samples investigated; AFM images of the uncapped structures are shown

In this work, we continue the study of photoelectric properties of the heterostructures with InAs QDs embedded in either the metamorphic In_0.15_Ga_0.85_As or conventional GaAs buffers, focusing on the effect of GaAs substrate and nearby MBE layers. In order to reach a clear understanding of the role of substrate and buffer layers, we considered the structures with bottom contacts on (i) the In_0.15_Ga_0.85_As buffer layer or (ii) the bottom GaAs substrate (see Fig. 1). Thus, depending on the bottom contact selection, the current flowed through (i) only the QDs and buffer layers and (ii) the complete structure including the substrates and their interfaces with MBE layers. The analysis of the results and calculations allowed us to provide an insight into the best design for light sensors on metamorphic QD structures.

## Methods

The structures were prepared by MBE on (001) semi-insulating (*si*) GaAs substrates. Substrates were *n*-type, with values of 3 × 10^7^ cm^−3^ residual carrier concentration, thickness of 500 μm, and a resistivity of 2 × 10^7^ Ω × cm. The metamorphic InAs/InGaAs QD structures consist of (i) 100-nm *n* ^*+*^-GaAs buffer layer grown at 600 °C, (ii) 300-nm thick *n* ^*+*^-In_0.15_Ga_0.85_As MB with *n* = 5 × 10^18^ cm^−3^ grown at 490 °C, (iii) 500-nm thick *n*-In_0.15_Ga_0.85_As MB with *n* = 3 × 10^16^ cm^−3^ grown at 490 °C, (iv) 3.0 monolayers (MLs) of self-assembled InAs QDs embedded in a 20-nm undoped In_0.15_Ga_0.85_As layer grown at 460 °C, (v) 300-nm *n*-In_0.15_Ga_0.85_As upper capping layer with *n* = 3 × 10^16^ cm^−3^ grown at 490 °C, and (vi) 13-nm *p* ^+^-doped In_0.15_Ga_0.85_As cap with *p* = 2 × 10^18^ cm^−3^ grown at 490 °C (Fig. 1). The growth rate was 1.0 ML/s, except for the QDs that were grown with a growth rate of 0.15 ML/s. The undoped layers are necessary to separate QDs from *n*-doped regions and, hence, to reduce the influence of non-radiative recombination centers, thus maximizing the QD light emission efficiency [30, 46]. The standard InAs/GaAs QD structures consist of (i) 300-nm *n* ^*+*^-GaAs buffer layer with *n* = 5 × 10^18^ cm^−3^ grown at 600 °C, (ii) 500-nm thick *n*-GaAs MB with *n* = 3 × 10^16^ cm^−3^ grown at 600 °C, (iii) 3.0 MLs of InAs QDs embedded in a 20-nm undoped GaAs layer grown at 460 °C, and (iv) 500-nm *n*-GaAs upper capping layer with *n* = 3 × 10^16^ cm^−3^ grown at 600 °C. The growth rate was 1.0 ML/s, except for the QDs that were grown with a growth rate of 0.15 ML/s.

Atomic force microscopy (AFM) images of the uncapped structures are shown in Fig. 1. By analysis of AFM data on similar structures, most frequent values of QD sizes were estimated to be 20 nm (diameter) and 4.9 nm (height) for metamorphic QDs and 21 nm (diameter) and 5.0 nm (height) for standard QDs [30, 31, 45].

For photoelectric measurements, circular 500-μm thick mesas were etched up on the structures down to bottom buffer *n* ^+^ layers; Au rectifying top contacts with a diameter of 400 μm and a thickness of 70 nm were then evaporated on the top of mesas. To obtain ohmic contacts on the bottom *n* ^+^-InGaAs and *n* ^+^-GaAs buffer layers, respectively, Au_0.83_Ge_0.12_Ni_0.05_ alloy was deposited at 400 °C for 1 min in nitrogen atmosphere. Thick indium ohmic contacts were made on the bottoms of substrates in other pieces of the same samples, in order to have measurements also with current flowing through the GaAs buffer and *si*-GaAs substrate. The ohmicity of the contacts has been verified by the *I*-*V* measurements, contacting to a piece of substrate; the current-voltage characteristics were found to be linear (data not shown).

Following the approach proposed in Ref. [47] and used in other works [48, 49], the thin *p* ^+^-InGaAs layer between the Au contact and the *n*-InGaAs layer was used to enhance the Schottky barrier height, since the structure obtained by the simple deposition of a metal on *n*-InGaAs exhibited a relatively low Schottky barrier height. Hence, the deposition of thin *p* ^+^-InGaAs layer enlarges the Schottky barrier height to be similar with that of Au-GaAs contact, maintaining resemblance of barrier profile for both the metamorphic and InAs/GaAs structures.

For structure and contact designing as well as understanding of the energy profile for both structures composed by the In_0.15_Ga_0.85_As or GaAs MBs, In(Ga)As QDs, undoped cap layer, and Au/AuGeNi contacts, the calculations were carried out using Tibercad software [50]. Band profiles were modeled in the drift-diffusion approximation, taking into consideration strain conditions, densities of traps related to defects at the InGaAs/GaAs interface region, depletion layers near contacts, and appropriate Schottky barrier heights. For the calculation of the metamorphic QD band profiles, sizes from AFM data were considered and strain effects were included, following an approach already validated in Refs. [42, 51]. The calculation of QD quantum levels is out of the scope of this paper, and QD modeling has been performed previously in Ref. [45]. In this work, however, we calculate band profiles of the whole heterostructure including the substrate.

Vertical photocurrent and PV spectra were measured in the 0.6 to 1.8 eV range using normal incidence excitation geometry at room temperature (RT) (300 K) and same light source intensity (1.5 mW/cm^2^). The photocurrent was measured using a current amplifier and direct current technique [10, 43–45], with 1 V bias. The current was measured as a voltage signal drop across a series load resistance of 100 kΩ (see the inset in Fig. 5). Photoluminescence (PL) excited at 532 nm was measured at 300 K. Some information concerning structures and methods is described in more detail in Ref. [45].

## Results and Discussion

### A. Photoelectric Characterization

**Fig. 2:**
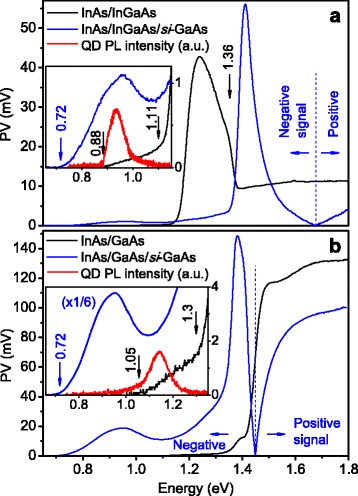
(Color online) room temperature PV spectra of the **a** metamorphic InAs/In_0.15_Ga_0.85_As and **b** InAs/GaAs QD structures; PV was measured contacted to only MBE layers [45] (black curves) and through the semi-insulating *si*-GaAs substrate (blue). The PV spectra measured through the *si*-GaAs substrate are inverted by sign of voltage below 1.68 and 1.44 eV respectively for **a** and **b**. Low-energy parts of the curves are given in the insets; the QD PL bands measured before [45] for both the structures are presented for the QD ground state energy positioning (red)

The PV spectra of both InAs/In_0.15_Ga_0.85_As and InAs/GaAs samples are presented in Fig. 2. Contacted to only the MBE layers, thick *n*-InGaAs, or *n*-GaAs buffers, the features of the spectra have been described elsewhere [45]. The spectrum threshold of the InAs/In_0.15_Ga_0.85_As at 0.88 eV is related to the ground state absorption in the QD ensemble, which corresponds to the onset of the QD band in the PL spectrum at RT measured earlier [45] (Fig. 2a). The metamorphic QD emission spectrum shows a wide band at 0.94 eV which is in the telecom range at 1.3 μm (0.95 eV), while the QD PL demonstrates a good efficiency, as it has been noted in earlier papers [30, 45, 46, 52]. The wide band of PV spectrum peaked at 1.24 eV and with edge at 1.11 eV is due to the carrier generation in the In_0.15_Ga_0.85_As MB and wetting layer (WL) including the way through the shallow levels. It should be added that the In_0.15_Ga_0.85_As bandgap calculated for MBE-grown layer is 1.225 eV [53], and the WL PL is observed at 1.2 eV [45]. Furthermore, a significant sharp fall above 1.36 eV is observed being caused likely by an indirect effect of the heavy doped GaAs buffer layer located outside the intercontact region that has been mentioned in Ref. [43]. The buffer layer is filled by numerous shallow levels and band non-uniformities originated from MBE growth defects and doping centers that redshift the interband absorption of GaAs [33, 46, 54, 55]. For the conventional buffer-contacted InAs/GaAs nanostructure, the onset at 1.05 eV of the PV spectrum in Fig. 2b originates from the QD ground state, as confirmed by the PL spectrum, while the sharp step at 1.3 eV can be related to the transitions in the WL [56]. The feature after 1.39 eV is obviously related to absorption of the doped GaAs upper buffer layer. A mechanism for this effect will be discussed in detail below.

As it is mentioned above, the sharp fall of PV signal above 1.36 eV in the InAs/In_0.15_Ga_0.85_As structure is related to *n* ^+^-GaAs bottom layer capping the substrate. To clear effects of the layers beneath the bottom AuGeNi contact on the photoresponse, we have studied photoelectric properties of the structures using an indium contact at the substrate back. Unlike the previous Au and AuGeNi contact geometry that allows for the unipolar PV, the bipolar signal has been observed for the structures contacted to the sample top and substrate back (Fig. 2). It is necessary to note that the PV sign changes along the photon energy axis, and in Fig. 2, the spectra of both the samples are inverted by sign of voltage underneath 1.68 and 1.44 eV for the InAs/In_0.15_Ga_0.85_As and InAs/GaAs QD structures respectively. Here, PV is considered to be positive when, as in the case of contact to the MBE layers, the positive potential is applied to the top Au contact while the negative one is applied to the bottom contact.

All the optical transitions mentioned above contribute to the PV signal of the structures in the substrate-top contact geometry. However, when measuring PV through the substrate, the signal onset for the metamorphic and conventional structures occurs at about 0.72 eV. The onset at 0.72 eV is attributed to the transition from the EL2 defect center located in *si*-GaAs substrate and related interfaces near 0.75 eV below the GaAs conduction band [57], taking into account the possibility of transition through the shallow levels of defects [46, 54, 55]. The aspects related to their location as well as the EL2 PC onset redshift have been discussed in detail elsewhere [10, 45]. As no signal underneath the QD-related bands was observed in the spectra of the samples contacted to the InGaAs or GaAs buffers (Fig. 2), we conclude that the substrate and related interfaces have no substantial influence on the properties of MBE-grown heterostructures.

**Fig. 3:**
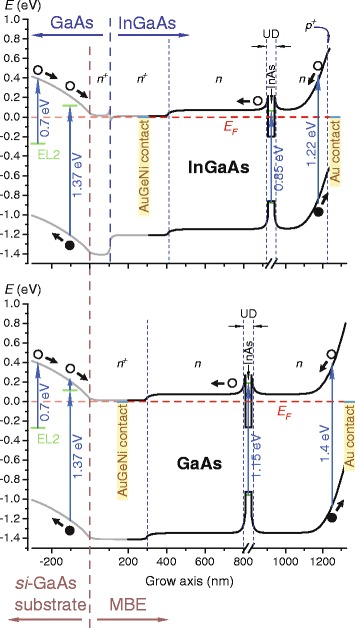
(Color online) calculated band profile in the metamorphic InAs/In_0.15_Ga_0.85_As (up) and pseudomorphic InAs/GaAs (down) structures, to explain the PV mechanism. The band bending of the deeper layers beneath the AuGeNi contact is indicated in gray. The optical transitions observed in the PV spectra are indicated by vertical arrows; bold arrows show drift directions of the optically excited charge carriers under the internal field (PV creation); *E*_F_ is Fermi energy. The calculations were carried out using Tibercad software [50]

To understand the appearance of the PV signal in our samples, one should look at Fig. 3 where we show the calculated band profiles along the growth direction. Detailed explanation of PV origin between the Au and AuGeNi contacts is given in the previous paper [45]. Summing up, the light-excited electrons (holes) drift predominantly toward the substrate (surface), giving a positive potential at the Au contact and a negative one at the AuGeNi contact.

Explaining the bipolar PV from the structures with the electrically active *si*-GaAs substrates, one can consider their calculated band structures in Fig. 3. Like before, the carriers generated in the top layers as well as in the QDs and WL might give “+” at the top and “−” at the substrate. The Fermi level in the semi-insulating substrate is located much lower than the one in the *n*-doped MBE layers. Therefore, the band bending near the *n* ^+^-GaAs/substrate interface is opposite to that in the rest of the MBE-grown structure (see the Fig. 3). Hence, the excitation in the *n* ^+^-GaAs layer and substrate (above 1.36 eV) gives an opposite PV signal to that from the QDs, WL, and buffers. The same applies to the excitation from EL2 defects (above 0.72 eV) of the GaAs substrate and especially EL2-like defects in *n* ^+^-GaAs/GaAs strained region [46, 57]. Contribution of the substrate and *n* ^+^-GaAs to the total PV signal is essentially stronger than that of the upper MBE layers, and the negative signal of PV is generally observed at lower excitation energies, while the impact of InGaAs layers and nanostructures appears as valleys on the respective spectral curves in Fig. 2. This is clearly seen by comparing the QDs, WL, and buffer spectral bands on the PV curves of the structures contacted to MBE buffers with the valleys in spectra of the substrate-top-contacted samples. For the higher energies, however, the excitation is absorbed closer to the sample surface not reaching the deeper MBE layers and substrate, which is the main source of negative signal. Hence, the PV signal becomes positive at larger energies. So, the presence of electrically active *si*-substrate leads to the competition between the spectral components related to the upper MBE-grown layers and the substrate-related defects and the *n* ^+^-GaAs absorption.

Otherwise, a similar characteristic feature above 1.35 eV has been observed by means of surface PV spectroscopy in a recent detailed study of *p*-doped InAs/GaAs QD and InAs/InGaAs dot-in-well structures based on *si*-GaAs substrates [58]. The drastic fall of the PV amplitude has been explained, unlike in our case, by different charge carriers generated below and above 1.35 eV. However, taking into account the drastic difference in the structures referred and present as well as specifics of the applied methods, we follow our interpretation of own results.

Based on the concept of the band bending below the AuGeNi contact, one can explain the sharp fall of PV signal in the buffer-contacted metamorphic InAs/InGaAs structure above 1.36 eV observed in Fig. 2a. This spectral feature is due to effect of the substrate and deepest MBE *n* ^+^-GaAs layer. Indeed, the electrons generated there move under the intrinsic field to the AuGeNi contact evoking an additional electric field there, herewith a barrier due to the band bending at InGaAs/GaAs heterojunction is obviously too low to be an essential obstacle for the charge carriers. This aligns the band bending in the upper layers, which directly contribute to the PV, and, hence, reduces the supply of the carriers photoexcited above the *n* ^+^-GaAs layer and, as a consequence, the total PV signal.

A small feature near 1.39 eV is observed in Fig. 2b in the spectrum of the pseudomorphic sample contacted to the MBE buffers, though a drastic fall of the signal like in metamorphic structure should be expected above 1.36 eV, taking into account a similar band bending near *n* ^+^-GaAs/substrate interface. Such a feature is not an attribute of only substrate and *n* ^+^-doped GaAs; such transitions were detected in In(Ga)As/GaAs QD structures based on *p*-doped [58] and undoped GaAs [10, 55]. These transitions obviously occur also in upper GaAs layers of our pseudomorphic structure, compensating mostly the negative effect of the near-substrate layers on the PV signal. As a result, only negligible influence of near-substrate layer can be observed on the black curve for InAs/GaAs sample in Fig. 2b rather than the fall in the curve of the metamorphic one originated from the deeper GaAs layers, despite a similar bipolar effect observed with direct participation of the substrate in PV formation.

The reason for the small feature after 1.39 eV in the spectrum of InAs/GaAs sample contacted to the MBE buffers can be different from the above-discussed for metamorphic InAs/InGaAs sample. In our opinion, it is due to the slight fall of signal caused by the absorption edge of the upper MBE-grown 500-nm thick GaAs buffer shading the QDs and WL which are more efficient contributors to PV at those photon energies. Indeed, electrons and holes generated in QDs and WL are carried to different sides and avoid recombination, unlike the volume generation, where recombination is much more probable. This is the main reason of effective detection of photocarriers coming from even a single layer of QDs and WL. Photons of higher energies are band-to-band absorbed in near-surface *n*-GaAs buffer layer and electrons escape to the sample volume away from the holes, leading to the sharp rise of PV above 1.4 eV. Correctness of the suggested reason for the 1.36 eV feature in the buffer-contacted InAs/GaAs structure rather than that assumed for metamorphic one is confirmed by studies of solar cells based on InAs/GaAs structures with the bottom contacts on the *n* ^+^-GaAs substrates [18, 24, 59], i.e., with a monotonous band bending through whole the sample from contact to contact. Their PV spectra reveal the same feature without a barrier related to the MBE-layer interface to the substrate. Furthermore, a narrow dip was observed in the same spectral range in the PC spectra of InGaAs/GaAs structures with lateral contact geometry and no intrinsic field [10, 55].

**Fig. 4:**
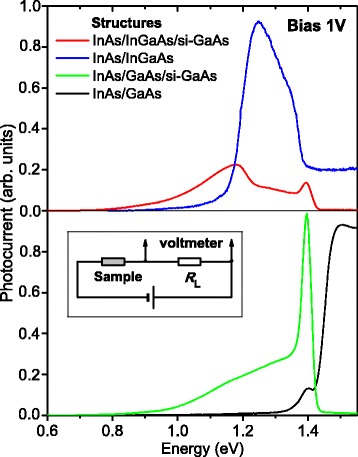
(Color online) room temperature photocurrent spectra of the metamorphic InAs/In_0.15_Ga_0.85_As/*si*-GaAs and conventional InAs/GaAs/*si*-GaAs QD structures. Inset: electric scheme of connecting the sample for PC measurements

The PC spectra of the structures obtained at 1 V bias directed like the intrinsic field in the upper layers of the structures (“−” at the top and “+” at the bottom contact) are presented in Fig. 4. The PC spectra for the structures contacted to the MBE layers are very similar to the PV ones in Fig. 2. The components from the QDs, WLs, InGaAs, or GaAs buffers as well as *n* ^+^-GaAs layer are observed at the same energies. Concerning the structures with the bottom contact on the s*i*-GaAs substrate, the PC spectra have thresholds near 0.72 eV related to the EL2 defect center absorption.

The features of PC spectra for the structures contacted to the MBE layers presented in Fig. 4 correspond mainly to those in the PV spectra in Fig. 2 considered above. Concerning the structures with the bottom contact on the s*i*-GaAs substrate with the EL2 center component, there is a competition between signal from absorption in the MBE layers and from EL2-related levels, as discussed above. However, the shapes of curves allow to conclude that no charge carriers excited within the layers above *n* ^+^-GaAs participate in PC; this is particularly relevant for the metamorphic QD structure spectrum. Obviously, the electrons do not reach the bottom because of the high potential barrier (see Fig. 3) induced by *si*-substrate. The substrate has too high resistance, and the main drop of applied bias occurs on it, hence, no barrier lowering occurs.

So, one can note that PV and photocurrent are negatively affected by the substrate-related *n* ^+^-GaAs layer: the absorption above 1.36 eV causes a drastic signal reduction. The main cause of the barrier below the AuGeNi contact is the *si*-GaAs substrate with a rather low positioning of the Fermi level resulting in the band bending opposite to that in the structure top. This is the only effect of the substrate observed in the PV at such contact geometry, and it manifests even at rather thick (400 nm) intermediate layer between the bottom contact and substrate.

### B. Substrate-Heterostructure Intermediate Layer Design Solutions

**Fig. 5:**
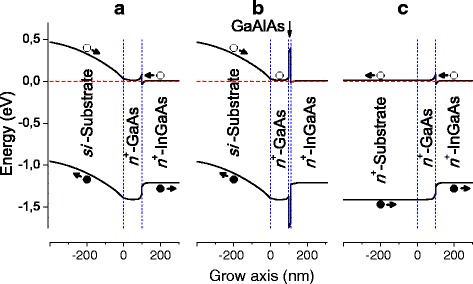
(Color online) calculated band profiles near In_0.15_Ga_0.85_As/GaAs interfaces of the metamorphic structure grown on a *si*-substrate with the *n* ^+^-GaAs layer thickness of **a** 100 nm (present sample), **b** 100 nm and a 10-nm thin Ga_0.3_Al_0.7_As barrier layer, and **c** structure like the present but grown on a *n* ^+^-substrate doped similar to the 100-nm thick *n* ^+^-GaAs layer above. The calculations were carried out using Tibercad software [50]

From a practical point of view, it can be concluded that such a design of InAs/InGaAs structure with *si*-GaAs substrate is not useful in the vertical light-sensitive device engineering, especially together with a relatively thin *n* ^+^-doped buffer, even when the contact configuration eliminates the current flow through the substrate. The space charge area formed in the *n* ^+^-GaAs/substrate interface region compels the charge carriers excited here to move oppositely to the ones excited in metamorphic structure, like in Figs. 3 and 5a, thus generating an opposite PV signal and reducing the total quantum efficiency of the sample.

Hence, for devices based on light absorption, a different structure design should be considered. We believe, such an improvement is necessary to be suggested because many research groups consider *si*-GaAs substrate as a basis for novel *p*-*n*-type both QD infrared photodetectors [11–13] and solar cells [15].

Simple thickening of the *n* ^+^-GaAs buffer under metamorphic structure seems to be not a very good idea. Though such a buffer could absorb more excitation quanta above 1.37 eV and shadow the interface and substrate below, its thickness has to be very high, because 800 nm of more narrow-bandgap InGaAs material above is insufficient to completely suppress the negative bipolar effects. Moreover, even a very thick *n* ^+^-GaAs buffer cannot exclude the negative effect of the EL2-like centers which are located mainly in the substrate and near their interface to the MBE layer. Nevertheless, as the charge carriers have a limited mean free path, thickening of the *n* ^+^-GaAs layer can weaken the induced negative field on the AuGeNi contact above.

A better improvement could be provided by growing a thin barrier layer for the electrons coming from substrate like it is shown at Fig. 5b. For calculations, a 10-nm thin undoped Ga_0.3_Al_0.7_As barrier layer has been chosen. Such barrier could strongly confine the electrons excited in the substrate within the *n* ^+^-GaAs layer. Similar high-ohmic layers grown by wide-bandgap materials as InAlAs, GaAlAs, and AlAs have been used in laser structures to avoid the charge carrier leakage from the active region of optoelectronic device [60]. However, for the case of GaAs-In_0.15_Ga_0.85_As based device, Ga_0.3_Al_0.7_As best matches due to the wide bandgap and small lattice mismatch between the epitaxial layer. Decreasing the carrier-induced field on the AuGeNi contact, it can suppress the negative effect of the substrate region on the photoresponse, especially in combination with increase in the *n* ^+^-InGaAs layer thickness.

Yet, a more optimal design of the vertical structures seems to be in use of a monotonous gradient of doping, including an *n* ^+^-doped GaAs substrate as it is proposed in Refs [14, 39, 40]. This design is the most efficient and at the same time simplest. If the substrate is doped similar to the capping *n* ^+^-layer or heavier, this causes the band bending depicted in Fig. 5c. Furthermore, an essential advantage of such a substrate could manifest itself in solar cell design. A low-resistive substrate allows for utilization of the configuration with the “–” contact on the sample bottom [24, 38–40, 59], non-shadowing the MBE structure from the sunlight.

## Conclusions

We have shown that photoelectrical characterization evidences a critical influence of the deep levels on the photoelectrical properties of vertical metamorphic InAs/In_0.15_Ga_0.85_As and pseudomorphic (conventional) InAs/GaAs QD structures in the case of electrically active *si*-GaAs substrate. Both nanostructures manifest a bipolar PV caused by a competition of the components originated from the oppositely sloped band profiles near the GaAs substrate and bottom MBE *n* ^+^-GaAs layer on one side and the rest of MBE-grown structure on the other side. An alternative contact configuration, which allows to avoid the current flow through the bottom layers, demonstrates the unipolar PV. The last configuration together with thick buffers on substrate strongly suppresses the influence of the photoactive deep levels originated from interfaces with the *si*-GaAs substrate on photoelectric properties of the nanostructures. However, a notable negative indirect effect of the substrate on the photovoltage and photocurrent signal from the InAs/InGaAs structure is observed when the excitation is absorbed in the substrate and near-substrate *n* ^+^-GaAs MBE layer. Analyzing the obtained results and the performed calculations, we have been able to provide insights on the design of metamorphic QD structures, which can be useful for the development of novel efficient photonic devices.

## Abbreviations

AFM: Atomic force microscopy
MB: Metamorphic buffer
MBE: Molecular beam epitaxy
ML: Monolayer
PC: Photoconductivity
PL: Photoluminescence
PV: Photovoltage
QD: Quantum dot
*R*_L_: Load resistance
*si*: Semi-insulating
WL: Wetting layer
